# Structural Characterisation of a Polysaccharide from Radix Ranunculus Ternati

**Published:** 2014

**Authors:** Xuefeng Huang, Yun Zhao, Xin Jin

**Affiliations:** a *Zhongshan Hospital, Xiamen University, Xiamen 361000, China.*; b *Medical College of Xiamen University, Xiang’an District, Xiamen 361000, China.*; c *Medical College of Xiamen University, Xiang’an District, Xiamen 361000, China.*

**Keywords:** Ranunculus ternatus Thunb, Polysaccharide, Ultrafiltration, Absolute configuration, Smith degradation, Methylation reaction, Anti-tumor activity

## Abstract

A water soluble polysaccharide, HB-1, with a molecular weight of 23,930, was isolated from radix Ranunculi ternati. by hot water extraction, ethanol precipitation, deproteination，ultrafiltration and gel-filtration column chromatography. Its sugar composition was determined by GLC as Glc, Ara, and Gal in a molar ration of 16.071: 2.722: 1. And the absolute configuration of Glc was identified as D. Smith degradation and methylation reaction showed the proportion of —^1^Glc (A) was about 16%, —^1^Glc^4^— (B) about 62%, (C) about 14%, and —^1^Gal^6^— (D) about 8%. The repetitive unit was likely composed of 3 As, 3 Cs, 13 Bs and 1 D. Together with the average molecular weight, it was predictable that HB-1 consisted of about seven of the repetitive unit. The inhibition activity of HB-1 on human glioma cell line SF188 was also measured, only to find it inactive.

## Introduction

Polysaccharides are present in large quantities in nature and have multiple applications such as immunomodulation(1), antioxidation(2), and antitumor(3), *etc*. Many of these bioactive polysaccharides are relatively non-toxic with no significant side effects (1-4). 

*Ranunc* *u* *lus ternatus* is a plant family of the *ranunculus*, which is accepted in the Chinese pharmacopoeia since 1990. *Radix* *Ranunculi ternati*, the root of *Ranunculus ternatus*, has been used for the treatment of faucitis, tuberculosis, neck scrofula, and breast cancer, *etc*. in traditional Chinese medicine. Pharmacological experiment indicated that the water extract of *Ranunculus ternatus* possessed not only inhibition on *staphylococcus aurers*, *pseudomonas aeruginosa*, but also remarkable inhibition on S_180_, S_37_ and Ec. In addition, its water extraction has been developed into hospital preparation in many forms, and proves to be very effective (5). Since the water-soluble part of *Ranunculus ternatus* Thunb are mostly sugars, polysaccharides in *Radix* *Ranunculi ternati* are worth investigating. 

During our investigation on the extracts from *radix* *Ranunculi ternati*, a polysaccharide (HB-1) was isolated and structurally elucidated. The isolation, purification, identification and its biological activities are reported in this paper.

## Experimental

*General*

GCMS spectra were taken on a Shimadzu GCMS-QP5050A spectrometer. GC analysis was carried out with GC9790 produced by Fuli company of China on columns of SE-30 (30 m × 0.32 mm × 0.5 μm) and OV-17 (30 m × 0.32 mm × 0.5 μm). HPLC was carried out using successive UV (Laballiance model 500) and evaporative light scattering (ELSD) (softa corporation model 200es ELSD) detector, series Ⅲ pump controlled by Ezchrom Elite Client/server(version 3.1.6), WYK-16b4 air compressor made by Dayin Instrument Factory in Tianjin, China, TOSOH TSK gel G4000PW_XL_ (7.8×30 cm) column, and HT-230A column oven made by Tianmeida Scientific Instrument Factory in Shenyang, China. Microplate reader was μQuant and was produced by Bio-tek Instruments Inc. ZDUF ultrafiltration machine was made by Zhongda Membrane Separation Instrument Factory in Hangzhou, China. L-cysteine methyl ester hydrochloride, N-Trimethysiylimidzole (TMSI), D-Glc and L-Glc were purchased from Sigma-aldrich company (St Louis, MO, USA). All other chemicals and reagents were analytical grade.

*Plant material*

*R*adix *Ranunculi ternati* was purchased from Bozhou county, Anhui province, China in 2004, and was identified by Qishi Sun, professor of Shenyang Pharmaceutical School, China. Voucher specimens were deposited at the Faculty of Pharmacy, Medical College, Xiamen University, Xiamen, China.

*Extraction and fractionation*

The dried powder of the roots of *Ranunculus ternatus* Thunb (2 Kg) was extracted with hot water for three times. The extraction was put together and concentrated in vacuum to 1000 mL, then precipitated with 70% ethanol. The precipitation was deproteinated by Sevage method (6) and ultrafiltrated by membranes of 6000 D and 10000 D, successively, through which two fractions with molecular weight of 6000-10000 (HA, with a yield of 22%) and ≥10000 (HB, with a yield of 13%), separately, were acquired.

*Purification*

**Figure 1:**
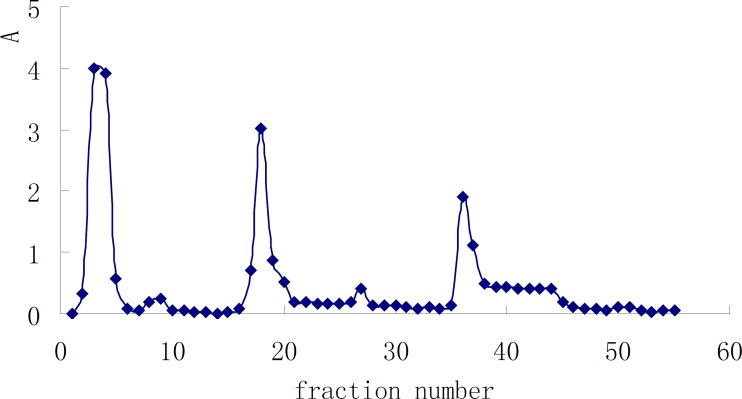
Elution profile of HB on DEAE Sephadex A-50

The crude polysaccharide, HB, was subjected to gel filtration on a 5L column of DEAE sephadex A-50, eluted at 0.5 mL/min with distilled water, followed by 0→1 M gradient of NaCl solution (7), and monitored using the phenol-sulfuric acid method (8), which gave several fractions according to their acidity (Figure 1). A subsequent purification step of these fractions, performed by sephadex G-100 gel-filtration after desalination with sephadex G-15, yielded a pure polysaccharide (HB-1) from fractions 2-5, and the yield was 0.03%. 

*Determination of molecular weight*

The average molecular weight of HB-1 was determined by high - performance liquid chromatography (HPLC) on a column of TSK gel G4000PW_XL_ (7.8×30 cm) monitored by ELSD, eluting with water at 0.5 mL/min, using purified dextran fractions with definite molecular weights as standards (9). 

*Sugar* *a* *nalysis*

HB-1 was hydrolyzed with 2 mol/L trifluoroacetic acid (TFA) at 110 ºC in a sealed tube for 2 h (10). After TFA was removed with N_2_, one part of the hydrolysate was analyzed by PC. The other part (2 mg) was derivatized with 0.4 mL hexamethyldisilazane (HMDS) and 0.2 mL trimethylchlorosilane (TMCS). The supernatant was subjected to GLC, which was performed with a SE-30 column (100 m*0.25 mm*0.25 μm) and an H_2_ flame ionization detector at a column temperature of 200 ºC, vaporizer temperature of 280 ºC, and detector temperature of 280 ºC (2), and the flow rate of the carrier gas (N_2_) was 2 mL/min.

*Absolute* *c* *onfiguration* *d* *etermination*

The polysaccharide was hydrolyzed with 2 mol/L TFA at 110 ºC in a sealed tube for 2 h and then dried with N_2_. Pyridine solution (1 mL) of the sugar (2 mg) was mixed with L-cysteine methyl ester hydrochloride (2 mg), and kept at 60 ºC for 2 h. After drying the mixtures with N_2_, N-Trimethysiylimidzole (TMSI, 0.2 mL) was added, and the warming at 60 ºC was continued for another hour. The reaction was ended by adding water (1 mL) into the container, and then the solution was extracted with cyclohexane (3 × 1 mL). Finally, the cyclohexane layer was collected and concentrated to 1 mL for GLC analysis, which was performed with an OV-17 column and an H_2_ flame ionization detector at a column temperature of 220 ºC, vaporizer temperature of 280 ºC , and detector temperature of 280 ºC (11).

*Periodate* *o* *xidation and smith* *d* *egradation*

HB-1(25.4 mg) was oxidized by 25 mL 15 mM sodium metaperiodate at 4 ºC in the dark. The production of formic acid and consumption of periodate were periodically determined by titration with 10 mM sodium hydroxide and colorimetry. After completion of the oxidation, the reaction was terminated by addition of ethylene glycol. Then KBH_4_ was added to each container to reduce the furfural. The mixture was dialyzed against distilled water for 48 h. Then the mixture was vacuum-dried and hydrolyzed with 2 mol/L TFA. The hydrolysate was acetylated and analyzed by GLC with an SE-30 column and an H_2_ flame ionization detector (FID) with column temperature program: 50 °C (5 min) to 235 °C (5 min.) at 10 °C/min , vaporizer temperature: 280 °C, and detector temperature:280 °C (12).

*Methylation*

To a solution of the dry polysaccharide (10 mg, 60 °C overnight on P_2_O_5_) in methyl sulfoxide (3 mL) was added powdered NaOH (sonic oscillation, 10 min) and methyl iodide (3 mL, dropwise, overnight). The mixture was stirred away from light at room tempreture for 1 h. The methylated polysaccharide was extracted with chloroform–water (1:1) for three times and the organic phase was dried with N_2_ stream. The methylated polysaccharide was hydrolyzed with 90% formic acid and 2 M TFA (1 mL), successively, for 6 h at 100 °C. Then the solution was dried with an N_2_ stream. The products were dissolved in water (1 mL) and reduced with 30 mg KBH_4_ (2 h at rt). Excess KBH_4_ was destroyed by drop wise addition of acetic acid (HAc) in ice. Then the mixture was treated with 3 mL of a solution of acetic acid: methanol (1:9) and dried with an N_2_ stream. This procedure was repeated four times. Successively, the mixture was repeatedly treated three times with methanol. The resulting alditols were acetylated with acetic anhydride (1 mL)–pyridine (1 mL) at 100 °C for 1 h, and then cooled and dried with an N_2_ stream. The alditol acetates were partitioned between chloroform and water (three times). The chloroform fractions were analyzed by GC–MS on a Shimadzu QP2010 GC–MS, equipped with a DB1capillary column (SGE, 25m*0.32 mm*0.52 μm) using a temperature programming of 150 °C (5 min) to 280 °C (10 min) at 10 °C/min. The carrier gas was Helium at a flow rate 1 mL/min (12). The EI mass spectrum was obtained by using an electron energy of 70 eV, an ionizing current of 0.2 mA and a source temperature of 150 °C. Masses were scanned from 41 to 400 amu during 1/2 s.

*Anti-tumor* *a* *ssay*

The human glioma cell line SF188 was grown as a monolayer in Dulbecco's Modified Eagle's Medium (DMEM) containing 10% fetal bovine serum (FBS). Cells were maintained in a humidified atmosphere of 5% CO_2_ in air at 37 ℃. Sensitivity of SF188 cells to polysaccharides was measured by 3-(4, 5- dimethylthiazol -2 –yl) -2, 5-diphenyltetrazolium bromide (MTT) assay. Briefly, exponentially growing cells were plated into 96-well plates (30,000 cells/well). After 24 h, serial dilutions of the drugs were added to the cells, which were incubated for 24 h. Then the cells were incubated with 10 μL of MTT (5 mg/mL) at 37 °C for 5 h. One hundred microliters of DMSO was added to solubilize the formazan crystals formed, and the optical densities at 570 nm were measured using a microplate reader.

## Results and Discusssion

**Figure 2:**
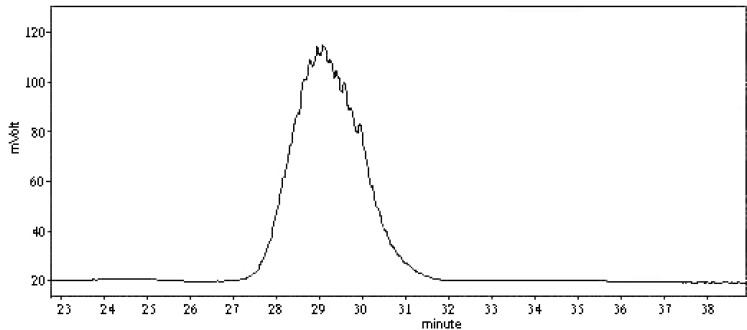
Elution profile of HB-1.

We now report the isolation and structural elucidation of a neutral polysaccharide from radix *Ranunculi *ternate. The crude polysaccharides HA and HB were prepared from this herb by hot water extraction, EtOH precipitation, deproteinization, and ultrafiltration. HB-1 was obtained from HB by DEAE Sephadex A-50 (Figure 1) and Sephadex G100 gel-filtration. Its purity was examined on TSKgel G4000PWXL column, see Figure 2 for the elution profile. The average molecular weight examined of HB-1 is 23,930, which is consistent with the result of ultrafiltration, so our job is a successful example of the use of ultrafiltration in the isolation of polysaccharides.

**Figure 3:**
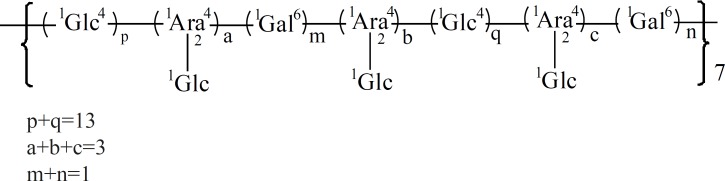
Repetitive unit of HB-1.

The lack of absorbance at 260 nm and 280 nm indicated that this polysaccharide contained no nucleic acid or protein. The sugar composition of HB-1 was determined by GC-MS as glucose (Glc), arabinose (Ara), and galactose (Gal) in a molar ratio of 16.071: 2.722: 1.000 and the absolute configuration of Glc were identified as D. The result of periodate oxidation and Smith degradation showed that the reaction products were mainly phycitol derived from (1→4) Glc and a few glycerine from (1→) Glc or Ara, (1→4) Ara, or (1→2) Ara. Methylation reaction showed that the proportion of —^1^Glc (A) was about 16%, —^1^Glc^4^— (B) about 62%, (C) about 14%, and —^1^Gal^6^— (D) about 8%. The proportion of A was almost the same as that of C, so came the presumption that there were 3 fragments of a attached to C, then C linked together with 13 Bs and 1 D, which was the unit that repeated to form the polysaccharide. The possible structure of the repetitive unit was shown in Figure 3. This unit had a molecular weight of 3350, according to the average molecular weight of the polysaccharide (23, 30), so it was predictable that HB-1 was composed of about seven of the repetitive unit.

Unfortunately, HB-1 did not possess the inhibition activity on human glioma cell line SF188. Studies show that, generally, both *Lanoderma lucidum* polysaccharides and lentinan with (1→3)-D-glucan structure have anticancer actions (13). Maybe this theory also applies to radix *Ranunculi *ternate polysaccharides. Since HB-1 consists of (1→2)- glucans, (1→4)- glucans and (1→6)- glucans instead of (1→3)- glucans, it is, in this way, explicable that it did not inhibit human glioma cell line SF188. 

But since the water extraction, which mostly consists of polysaccharides, of radix *Ranunculi *ternate has been used as hospital preparation to treat breast cancer and proves to be very effective, there must be a substance, or some substances working together to support this therapeutic effect, which needs further investigation. 
